# Fluorescence and phosphorescence of tetracarboxylated ZnPc–perylene diimide organic systems in highly acidic media

**DOI:** 10.1039/d5ra06479a

**Published:** 2026-02-25

**Authors:** Vadim Furtuna, Ion Lungu, Tamara Potlog, Alexandrina Druta, Anton Airinei

**Affiliations:** a Laboratory of Organic/Inorganic Material for Optoelectronics, Moldova State University A. Mateevici 60 str. 2009 Chisinau Moldova; b Laboratory of Physical Chemistry of Polymers Petru Poni Institute of Macromolecular Chemistry Iasi 700487 Romania

## Abstract

This study presents a comprehensive analysis of the photophysicochemical properties of zinc(ii) 2,9,16,23-tetracarboxy-phthalocyanine (TcPcZn), *N*,*N*′-bis(3-pentyl)perylene-3,4,9,10-bis(dicarboximide) (EP-PDI), and their mixed systems in various TFA/H_2_O solvent mixtures. Key aspects discussed include molecular arrangement, intra- and intermolecular interactions, protonation effects on absorbance and aggregation, light-induced partial deprotonation of TcPcZn, and Förster Resonance Energy Transfer (FRET) effects on excited-state lifetimes. Due to strong solvent quenching, the singlet-state lifetimes of EP-PDI (1.07 and 2.91 ns), TcPcZn (0.67 and 3.79 ns), and their TcPcZn : EP-PDI mixture (0.64 and 3.96 ns) were measurable only at high concentrations (*C*_M_ ≥ 0.110 mM). Both individual compounds and their blends exhibit promising triplet-state lifetimes, which are crucial for processes relying on long-lived excited states, such as photodynamic therapy and optoelectronic applications. In this context, particular attention is given to their ability to sustain room-temperature phosphorescence (RTP) : TcPcZn – 8.93 µs, EP-PDI – 10.18 µs, and TcPcZn : EP-PDI – 9.41 µs. Several strategies are proposed to further optimize these parameters and enhance RTP quantum yields.

## Abstract 2

Co-assembly of TcPcZn and EP-PDI in acidic TFA/H_2_O enables tunable excited-state lifetimes and room-temperature phosphorescence, providing design principles for efficient organic photosensitizers in photodynamic therapy and optoelectronics.

## Graphical abstract

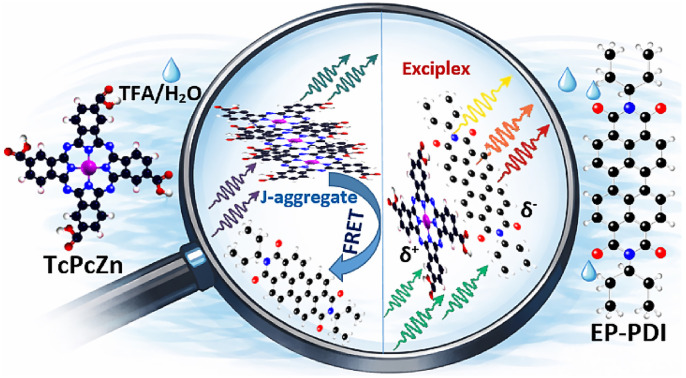

## Introduction

Zinc(ii) tetracarboxyphthalocyanine (TcPcZn) exhibits distinctive photophysical properties, including pronounced Q-band absorption in the 695–765 nm region, alongside both fluorescence and room-temperature phosphorescence (RTP).^1^ Notably, TcPcZn-based composites—such as ZnPc(COOH)_4_/chitosan/Fe_3_O_4_—have demonstrated triplet-state lifetimes of up to 12.3 µs and a triplet quantum yield of approximately 0.56, confirming their potential for applications in photodynamic therapy (PDT).^2^ Furthermore, owing to its unique molecular architecture, TcPcZn holds significant promise for applications in bioimaging, optoelectronics, and chemical sensing.^3,4^ However, fine-tuning the excited-state behavior through mole-cular engineering remains crucial for enhancing its functional performance.

A key strength of TcPcZn lies in its peripheral carboxyl groups, which significantly enhance its chemical versatility and ability to form supramolecular assemblies or conjugates with biocompatible materials. For instance, in a study involving mo-nomeric TcPcZn loaded onto upconversion nanoparticles and coated with a hyaluronic acid (HA) gel, this photosensitizer exhibited effective near-infrared (NIR)-triggered photodynamic activity *in vitro*.^5^ While the carboxylate functionality enables targeted interaction with biomolecules such as HA or amino-modified surfaces, it also facilitates electrostatic and hydrogen bonding-driven self-assembly, essential for building donor–acceptor nanostructures.

Nevertheless, the same study also noted that TcPcZn tends to aggregate in aqueous environments, leading to decreased singlet oxygen yield due to excitonic quenching. This under-scores the need to modulate aggregation and electronic interac-tions *via* strategic co-assembly or encapsulation approaches.

A promising strategy involves combining TcPcZn with electron acceptors capable of modulating its optical characteristics and excited-state dynamics. *N*,*N*′-bis(3-pentyl) perylene-3,4,9,10-bis(dicarboximide) (EP-PDI) is selected as an efficient acceptor due to its high electron affinity, photostability, and well-defined absorption features in the visible range.^6,7^ Co-assembling ZnPc(COOH)_4_ with EP-PDI in trifluoroacetic acid/water (TFA/H_2_O) mixtures at varying volume ratios is expected to promote favorable non-covalent donor–acceptor interactions, primarily *via* π–π stacking and electrostatic/hydrogen-bonding effects, which can enhance light absorption and emission properties.

This study investigates the photophysicochemical behavior of these mixed systems, focusing on how molecular interactions influence fluorescence intensity and RTP lifetimes. The results aim to support the development of ZnPc-based donor–acceptor architectures for advanced photonic applications.

## Materials and characterisation

TcPcZn was synthesised and purified according to the procedure reported previously.^2^ TFA (99.9%) and EP-PDI (98%) were purchased from Merck KGaA (Darmstadt, Germany) and used without further purification. Redistilled water was used for the preparation of all solutions.

UV-vis spectra were recorded using a Lambda 25 spectrophotometer (PerkinElmer Inc., Shelton, CT, USA) over the range of 200–1200 nm in 10 mm quartz cuvettes. Steady-state fluorescence emission spectra of TcPcZn, EP-PDI, and their mixtures were recorded on an LS55 spectrometer (PerkinElmer Inc., Shelton, CT, USA) equipped with double-grating excitation and emission monochromators, with a 150 W Xe lamp as the excitation source. Excitation wavelengths were selected at 360, 390, 400, 490, 660, and 705 nm, and emission spectra were collected in the 410–850 nm range with a step size of 1 nm. Slit widths were varied between 5 and 15 nm depending on signal intensity, no optical filters were used, and all spectra were corrected for the detector response. Independent repetitions showed consistent spectral shapes and intensities within an experimental error of ±5%. Fluorescence lifetimes were determined using a time-correlated single photon counting (TCSPC) system. Time-resolved fluorescence spectra were recorded with an FLS980 spectrometer (Edinburgh Instruments, Livingston, EH54 7DQ, Oxford, UK). All measurements were carried out at room temperature (295 ± 1 K). pH values were measured using a pH meter (ISOLAB Laborgeräte GmbH, Germany).

## Experimental

Initially, 2 mg of TcPcZn and 2 mg of EP-PDI were dissolved separately in 4 mL of a TFA/H_2_O solvent mixture (1 : 1 by volume) at room temperature. The molar concentrations of these solutions were analytically calculated and was obtained *C*_M_(C_36_H_16_N_8_O_8_Zn) = 0.663 mM and *C*_M_(C_34_H_30_N_2_O_4_) = 0.942 mM. Each of these solutions was subsequently diluted with 8 mL of H_2_O and the ZnP and PD solutions were thus obtained. From these solutions, through a series of dilution operations with water at 25 °C, the solutions marked ZnP1, ZnP2, ZnP3 and PD1, PD2, PD3 were obtained. The molar concentrations, pH values, and volumetric ratios of the solvents in each solution are indicated in Table 1.

**Table 1:** pH values, solvent volumetric ratios(*φ*), and molar concentrations of TcPcZn and EP-PDI solutions(*C*_M_)

Reference solution	*C*_M_, mM	*φ*(TFA), %	*φ*(H_2_O), %	Estimated pH (±0.02)	Measured pH (±0.01)
ZnP	0.221	16.67	83.33	0.04	0.03
ZnP1	0.055	4.175	95.825	0.48	0.46
ZnP2	0.088	6.67	93.33	0.34	0.32
ZnP3	0.110	8.335	91.665	0.28	0.26
PD	0.314	16.67	83.33	0.04	0.04
PD1	0.078	4.175	95.825	0.49	0.47
PD2	0.126	6.67	93.33	0.35	0.33
PD3	0.157	8.335	91.665	0.29	0.27

## Results and discussion

**Fig. 1:**
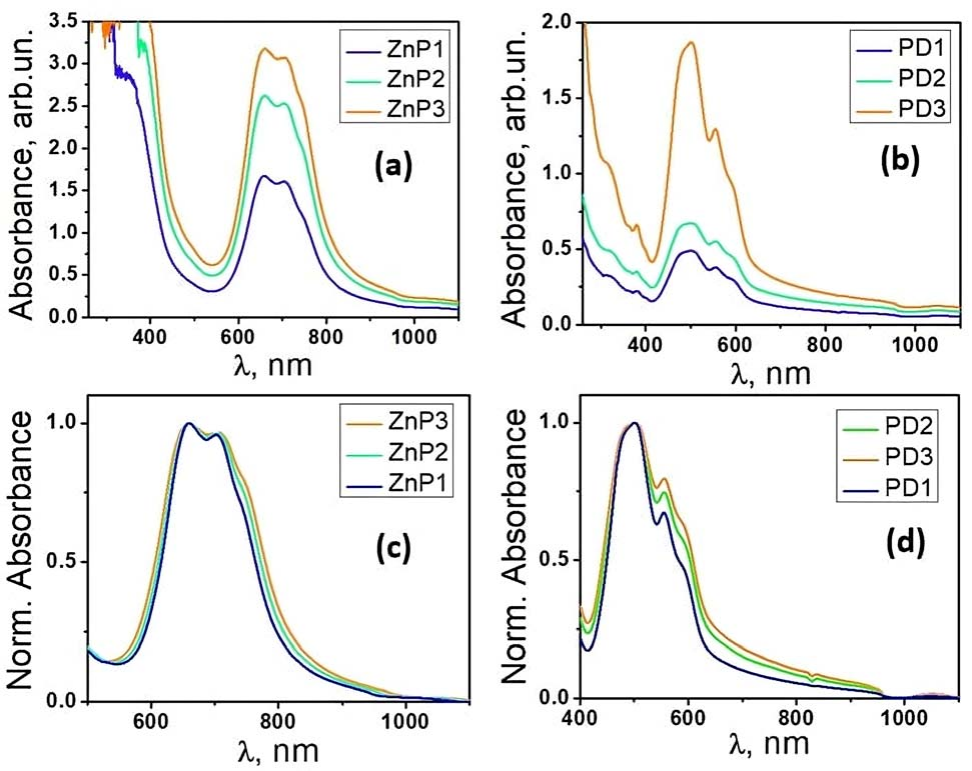
Absorbance spectra of (a) TcPcZn in ZnP1–ZnP3 solutions and (b) EP-PDI in PD1–PD3 solutions; normalized absorbance spectra of (c) TcPcZn and (d) EP-PDI recorded under identical conditions.

The UV-vis absorption spectra of TcPcZn solutions are presen-ted in Fig. 1a. The graphs primarily reveal the presence of the main absorption bands B and Q, specific for TcPcZn, as well as the increase in absorbance with increasing solution concentra-tions. As can be seen from the absorbance curves, the Soret band is approximately 30 nm red-shifted,^8^ compared to its posi-tion in the absorbance spectra of TcPcZn solutions in polar a-protic solvents and possesses an irregular shape below 350 nm.The explanation lies in the fact that this band is slightly solvatochromic because the electronic transition involved (π → π*) is, to a certain extent, sensitive to solvation effects. Water, being highly polar, tends to stabilize the excited state, leading to a red shift of the Soret band. TFA, while also polar, has a different dielectric constant and causes a different shift, but the combination of these solvents leads to a more pronounced solvatochromic effect than either solvent alone, likely enhancing the bathochromic shift of the Soret band. It is well known that water is relatively transparent in the UV-vis spectrum above 200 nm, while TFA has more significant absorp-tion in the UV region, especially in the range of 200–250 nm, due to the carbonyl (C=O) and C–F bonds in the molecule, which can participate in electronic transitions that absorb light in the UV.^9^ Importantly, the irregular shape of the Soret band is not an instrumental artifact: it persists across a dilution series well within the linear range of the spectrometer and after baseline subtraction using the TFA/H_2_O blank. Based on these considerations, we conclude that the irregular shape of the Soret band is most likely due to interferences caused by the presence of TFA species in the analyzed systems (which can involve protonation and aggregation effects), as well as from spectral overlap with the TFA/H_2_O background.

As expected for a solution obtained by dissolving a function-alized derivative of ZnPc in a mixture of protic solvents, one of which is acidic,^10,11^ the Q absorption band splits and exhibits two peaks at 660 nm and 706 nm, respectively. As is known, the splitting of the Q absorption band of ZnPc (zinc phthalocyanine) and functionalized ZnPc in acidic solvents is primarily due to the protonation of the molecule and the resulting changes in its electronic structure and symmetry.^10–13^ In our systems, part of the hydrogen cations, obtained through the electrolytic dissociation of TFA, protonate the carboxyl groups and the non-pyrrolic nitrogen atoms of the TcPcZn.^13^ Due to the presence of a larger number of water molecules, because of serial dilution, protonation is most likely achieved with the help of hydronium cations (H_3_O^+^).

**Fig. 2:**
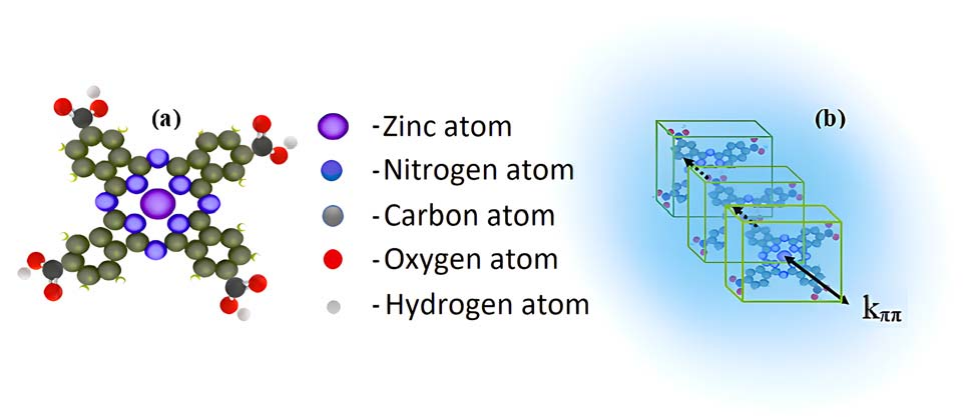
(a) 3D structure of the TcPcZn molecule; (b) π–π stacking interactions between TcPcZn molecules in an H_2_O/TFA mixture.

At the same time, we also observe that the Q band broadens with increasing concentration of TcPcZn and TFA in solution (Fig. 1c) and in our case is approximately twice as wide as for TcPcZn dissolved in sulfuric acid,^14^ or in other solvents.^15,16^ We believe that this is due to the aggregation of solute molecules, which intensifies with increasing of their concentration and that of TFA. When the carboxyl groups are protonated, the TcPcZn molecules are less likely to be repelled from each other and may be more likely to aggregate, as there is no longer significant repulsive force from the carboxylate anions.^17^ Furthermore, protonation of the carboxyl groups diminishes their electron-withdrawing effect,^11,18^ leading to a more neutral and less polar surface on the TcPcZn molecules, which may facilitate stronger π–π stacking interactions (Fig. 2b), promoting primarily H-type aggregation.

On the other hand, due to reduced charge repulsion and stabilizing hydrogen bonding, protonation in our specific case of ZnPc-(COOH)_4_ in acidic aqueous solution can likely promote the formation of J-aggregates at low to intermediate concentrations. Protonated carboxyl groups can engage in hydrogen bonding with molecules of the mixed solvent, particularly water, helping to stabilize TcPcZn molecules in a less aggregated state at the early stages. As the concentration of TcPcZn increases (from ZnP1 to ZnP3), intermolecular interactions intensify, leading to aggregation. The TFA co-solvent can also participate in hydrogen bonding with the carboxyl groups, further stabilizing the protonated TcPcZn and promoting aggregation under favorable conditions. This is further supported by the slight bathochromic shift of the Q-band, suggesting that, alongside face-to-face stacking, head-to-tail molecular organization characteristic of J-type aggregates also occurs.^19^

**Fig. 3:**
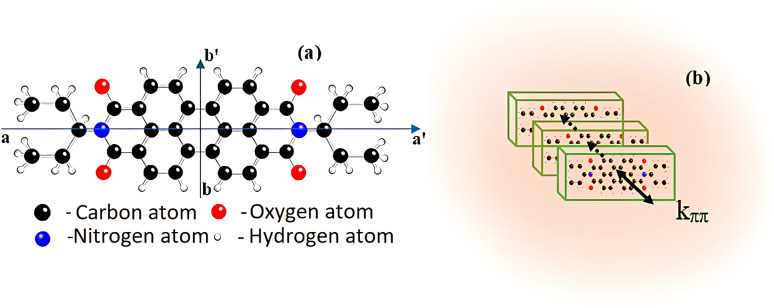
(a) 3D structure of the EP-PDI molecule with its long (a, a′) and short (b, b′) axes; (b) H-aggregation of EP-PDI in an H_2_O/TFA mixture.

Investigating the UV-vis absorption spectrum of EP-PDI in tetrahydrofuran,^20^ Samir Maity *et al.* reported that it can be divided into two well distinct regions, located at 410–550 nm and 255–370 nm, and this spectrum corresponds to two types of electronic transitions.^21^ Similarly, the UV-vis absorption spectrum of EP-PDI in a H_2_O/TFA mixture (Fig. 1b) is divided into two regions. The short-wavelength band is blue-shifted and extends below 260 nm, with a maximum tendency around 250 nm, but this portion is not fully shown in Fig. 1b due to strong absorption by the TFA/H_2_O solvent mixture, which partially masks the solute contribution. For clarity, only the region above 260 nm is presented, where the absorption features of EP-PDI are more clearly distinguishable from the solvent background.The peaks of this band correspond to transitions between different vibronic levels along the short axis of perylene chromophore (Fig. 3a).^20^ We assume that the hypsochromic shift of this absorption band arises from complex intermolecular interactions, both among the chromophore molecules themselves and between the chromophores and the species present in the solvent mixture.

The π–π interactions between EP-PDI molecules promote face-to-face stacking (Fig. 3b), resulting in formation of H-aggregates^19^ and an increase in the HOMO–LUMO gap at higher excited states.^22^ In addition to this, certain interactions in lower pH systems could provide transient protonation of the carbonyl oxygens of the perylene bisimide. Protonation typically introduces a positive charge to an oxygen atom (in the imide group), which affects the electron density distribution in the molecule. This inevitably leads to changes in the highest occupied molecular orbital (HOMO) and lowest unoccupied molecular orbital (LUMO) energies, which directly influence the absorption properties.^23^ Furthermore, despite the fact that the carbonyl groups of the dicarboximide moiety exert an electron-withdrawing effect on the nitrogen atom (which is also *N*-alkylated with a 3-pentyl group), thereby decreasing its basicity through resonance delocalization of the lone pair and making it less capable of attracting and accepting a proton, transient protonation of the imide nitrogen may still occur in the presence of TFA due to its strong acidity (such protonation becomes plausible at pH< 1). Water can also play a role in protonation, but its influence would likely be secondary compared to TFA. In fact, water can help stabilize protonated species through hydrogen bonding, but the key factor here is that TFA provides a strongly acidic environment. The protonation of the carboximide groups makes them more hydrophilic, and that could hinder the aggregation because water molecules would likely solvate the hydrophilic parts of the EP-PDI, potentially shielding the hydrophobic perylene units from close proximity required for strong π–π stacking interactions. TFA is a polar solvent, but has a low dielectric constant (8.55 at 25 °C) compared to water (∼80.1 at 25 °C),^24^ which means it might reduce the solvation of EP-PDI hydrophobic parts (alkyl chains) and thus, in lower pH systems, aggregation is more likely driven by hydrophobic forces rather than π–π stacking, which might be more disrupted by TFA.

Judging by the width and slight redshift of the second absorption band (Fig. 1b), we conclude that, in our EP-PDI/TFA/H_2_O systems, in addition to the predominant face-to-face (H-type) stacking, some head-to-tail or slipped aggregation also occurs, leading to J-type molecular aggregates.^19^ As can be seen, this band, spanning 412–650 nm with a maximum at 502 nm (corresponding to transitions along the long axis of the perylene chromophore, Fig. 3a), is broader and slightly red-shifted compared to EP-PDI in other polar and non-polar solvents reported in the literature.^20,25,26^ Our findings are consistent with previous studies on EP-PDI,^27,28^ which show that its π–π stacks can adopt an oblique orientation. This stacking geometry therefore supports the formation of aggregated species and, as will be shown later, several features observed in the emission spectra.

**Fig. 4:**
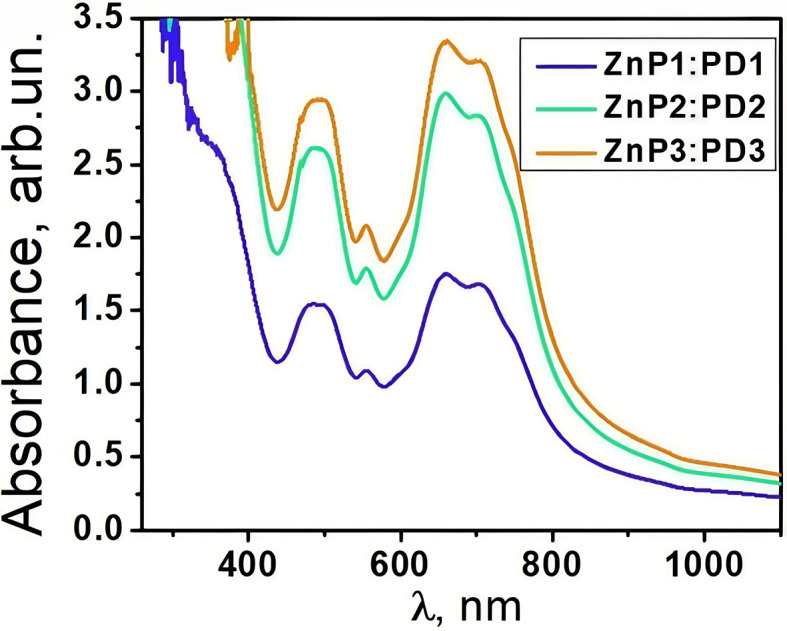
UV-vis absorption spectra of the TcPcZn : EP-PDI mixtures in the TFA/H_2_O mixed solvents with varying volume ratios.

Fig. 4 shows the UV-vis absorption spectra of the TcPcZn : EP-PDI mixtures prepared by combining, in equal volumes, the solutions listed in Table 1. A comparison with the spectra in Fig. 1a and b reveals that the peak positions associated with each component remain practically unchanged. At the same time, even though the concentrations of the solute species decrease by a factor of two upon mixing, the absorbance intensities of both TcPcZn and EP-PDI increase noticeably. For the ZnP1 : PD1 mixture, the absorbance of TcPcZn increases by approximately 0.1 units, while that of EP-PDI increases by about threefold. In the ZnP2 : PD2 mixture, the TcPcZn absorbance increases by ∼0.4 units and the EP-PDI absorbance by about fourfold, whereas for the more concentrated ZnP3 : PD3 mixture, the absorbance increases by ∼0.3 units for TcPcZn and by ∼1.6-fold for EP-PDI.

**Fig. 5:**
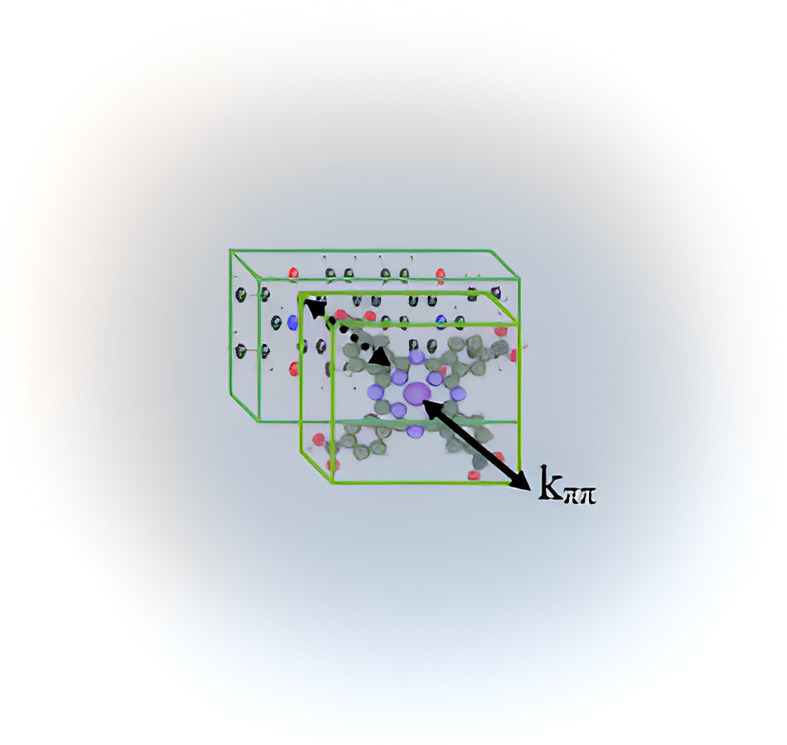
π–π stacking interactions between TcPcZn and EP-PDI molecules in an H_2_O/TFA mixture.

Such an increase in absorbance upon dilution contradicts the Beer–Lambert law and suggests the formation of supramolecular aggregates or donor–acceptor complexes between TcPcZn and EP-PDI.^29–31^ Although the concentrations of the individual donor (TcPcZn) and acceptor (EP-PDI) species are halved, aggregated species may display stronger absorption than the isolated molecules. Axial coordination can be excluded because EP-PDI lacks coordinating groups for Zn^2+^, and aggregation is therefore most likely driven by non-covalent π–π interactions between the components (Fig. 5), as both TcPcZn and perylene bisimide possess extended aromatic systems.

However, the increase in absorption intensity alone cannot be considered compelling evidence for π–π stacking, as different aggregation modes may affect oscillator strengths in various ways. A stronger indication of intermolecular interactions arises from the broadening of the absorption bands and the decreased vibronic structure in Fig. 4 compared to Fig. 1. These spectral features are consistent with enhanced electronic coupling and increased structural disorder in aggregated systems. Although light scattering may contribute slightly to the apparent intensity increase, the characteristic absorption bands of both components remain clearly distinguishable, supporting the conclusion that band broadening and the reduction of vibronic structure—rather than changes in intensity—provide the most compelling evidence for aggregation in these mixtures.

**Fig. 6:**
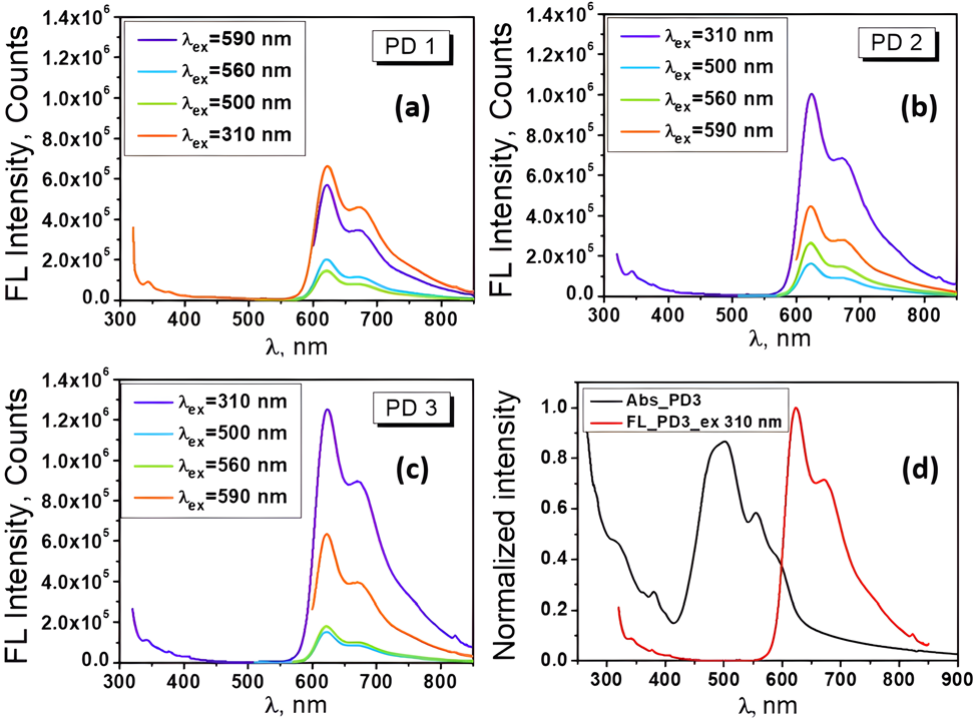
(a–c) Fluorescence spectra of EP-PDI in PD1–PD3 solutions excited at different wavelengths; (d) normalized absorbance and emission spectra of EP-PDI upon excitation at 310 nm.

Examining the fluorescence spectra of our EP-PDI/TFA/H_2_O systems (Fig. 6a–c) in correlation with their absorbance spectra shown in Fig. 1b, we observe a broad emission band with the peak at ∼623 nm and a shoulder on the right side at ∼672 nm. Also, as shown in Fig. 6d, which presents the normalized absorbance and emission spectra of EP–PDI upon excitation at 310 nm, both the peak ratios and the shapes of the spectra do not reflect a true mirror-image relationship, with the fluorescence spectrum appearing as a shifted version of the second band of the absorption spectrum. The Stokes shift is estimated to be ∼118 nm, which is unusually large for EP-PDI and can be attributed primarily to the highly polar and protic TFA/H_2_O mixed solvent (Lippert–Mataga effect).^20^ The absence of a mirror-image relationship suggests that, in addition to solvent effects, multiple photophysical processes may occur in the excited state of EP-PDI, including aggregate or excimer emission and deprotonation of the transiently protonated carbonyl oxygens of the perylene bisimide upon excitation, highlighting the complexity of its excited-state behavior.

**Fig. 7:**
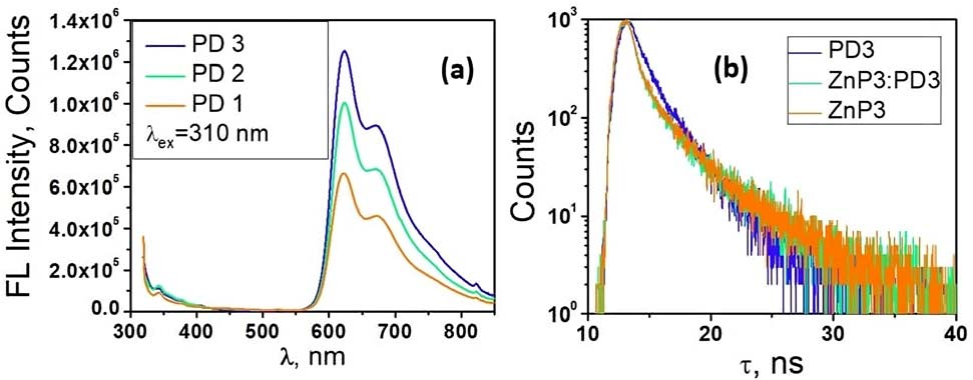
(a) Fluorescence spectra of EP-PDI in PD1–PD3 solutions upon excitation at 310 nm; (b) fluorescence lifetime spectra of TcPcZn, EP-PDI, and the TcPcZn : EP-PDI self-assembled system in a TFA/H_2_O mixed solvent.

These spectra also highlight the dependence of the fluorescence intensity on the wavelength of the excitation radiation, as well as on the concentration of the analyte in solution. As can be seen, the fluorescence intensity of the chromophore increases significantly with increasing its concentration in the analyzed solutions and this dependence is rendered more clearly in Fig. 7a. Here, we see that the shape of the spectra remains the same, but at higher concentrations, the emission band between 560-800 nm becomes markedly more intense.

At first glance, the right shoulder of the emission band could be attributed to an internal charge-transfer (ICT) state, which is known to form rapidly after excitation.^32^ However, no evidence has been reported for the occurrence of an intramolecular charge-transfer state in EP-PDI upon excitation, even in highly polar solvents. A closer analysis of the emission spectra suggests that the emission feature on the right side, around 672–700 nm, can be attributed primarily to the vibronic progression of the perylene core fluorescence rather than to a distinct excimer-related band, as also discussed for similar perylene derivatives.^33^ This interpretation is supported by the relatively small intensity of this feature, its nearly constant spectral position and ratio relative to the main peak (∼623 nm), and the characteristic vibronic spacing of approximately 1170 cm^−1^, which is consistent with the expected vibronic transitions of perylene-based chromophores. Nevertheless, considering the unusually large Stokes shift observed for this system and the highly polar, protic TFA/H_2_O environment, a minor contribution from emissive aggregates or excimer-like species cannot be completely excluded. There are reports in the literature that EP-PDI can form two types of excimers, *i.e.*, Y-excimer and E-excimer, depending on the relative orientation of two approaching molecules.^20,34,35^ It is also well known that the coexistence of monomers and excimers in solution or the solid state is a common reason for observing two distinct fluorescence lifetimes in decay measurements.^20,36,37^

For the PD3 solution, the measured lifetimes upon excitation at 500 nm are presented in Table 2. In this context, we assume that the major component, with a fluorescence lifetime of approximately 1.07 ns, arises from monomeric decay from the singlet excited state of EP-PDI. In highly acidic media such as TFA/H_2_O, the fluorescence of perylene diimide derivatives is strongly quenched due to protonation and increased nonradiative deactivation, which significantly shortens the lifetime compared to values typically observed in neutral organic solvents. The longer component, with a lifetime of 2.91 ns, is attributed to E-excimer emission of EP-PDI. Therefore, a biexponential fit was required to analyze the fluorescence decay curve (Fig. 7b).

**Table 2:** Excitation wavelengths (*λ*_ex_), fluorescence lifetimes (*τ*_1_ and *τ*_2_), relative contributions (% Rel), and fluorescence quantum yields(*Φ*)

Samples	FL					
	*λ*_ex_, nm	*τ*_1_, ns	*τ*_2_, ns	Rel *τ*_1_, %	Rel *τ*_2_, %	*Φ*, %
PD 3	500	1.07	2.91	61.48	38.52	6.02
PD 3	355					5.32
ZnP3 : PD3	660					1.99
ZnP3 : PD3	355	0.64	3.96	64.33	35.67	1.87
ZnP3	660					9.51
ZnP3	355	0.67	3.79	62.69	37.31	—

The reduced fluorescence quantum yield (*Φ*) is due, on the one hand, to H-aggregation,^38,39^ which facilitates the formation of E-type excimers, and, on the other hand, to strong solvent quenching. Following excitation, the dipoles of H_2_O and TFA interact with that of EP-PDI (µE) and can reorient or relax around it, which strongly lowers the energy of the excited state of the fluorophore. Most likely, due to the strong quenching caused by the mixed solvent, the lifetimes of the EP-PDI could not be recorded in the more diluted PD1 and PD2 systems.

**Fig. 8:**
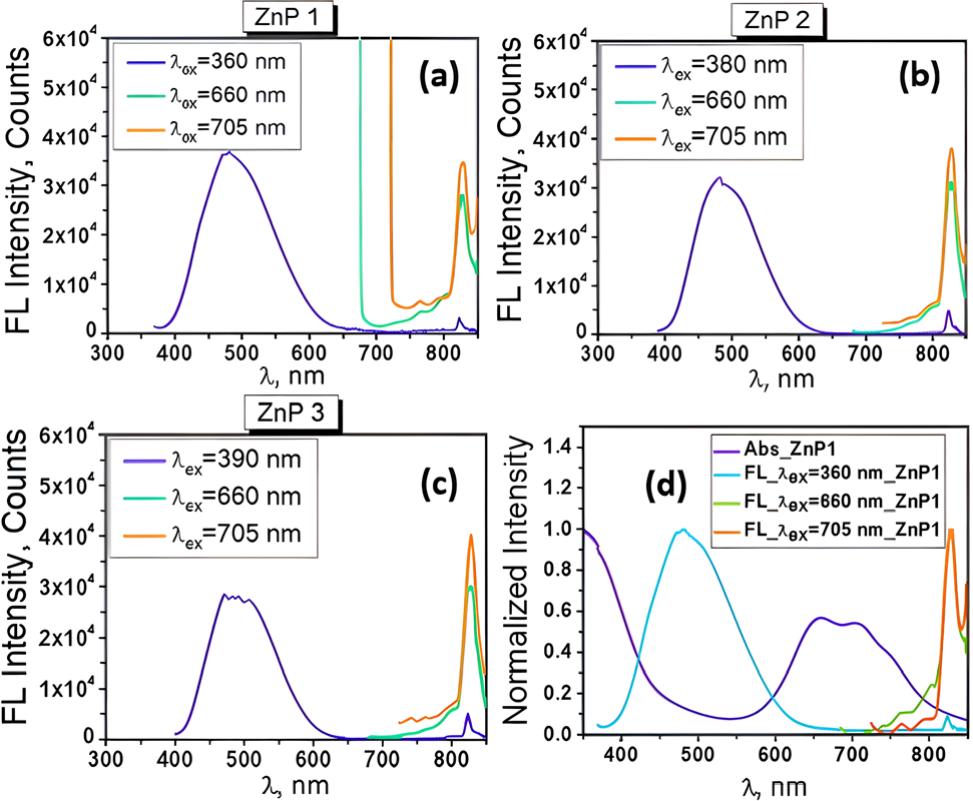
(a–c) Fluorescence spectra of TcPcZn in ZnP1–ZnP3 solu-tions under excitation at different wavelengths; (d) Normalized absorption and fluorescence spectra of TcPcZn in ZnP1 solution.

The fluorescence spectra of the TcPcZn in TFA/H_2_O mixtures are presented in Fig. 8. A comparison with the corresponding absorption spectra (Fig. 1a and 8d) suddenly reveals a notably weak, or nearly absent, mirror-image relationship between the excitation and emission profiles. Upon excitation of all ZnP1–ZnP3 solutions at *λ*_ex_ = 660 nm and 705 nm, the emission spectra consistently exhibit a band in the 750–850+ nm range, with a maximum at approximately 829 nm, corresponding to the characteristic Q-band fluorescence of TcPcZn, which arises from the *S*_1_ → *S*_0_ transition. Due to instrumental limitations, fluorescence signals above 850 nm could not be recorded.

Remarkably, excitation of the TcPcZn/TFA/H_2_O systems at wavelengths below 400 nm results in the appearance of a new emission band in the 400–650 nm region (with a peak centered at ∼481 nm) and a decrease in intensity of the band located between 750-850+ nm. These unexpected emission features are of particular interest and suggest the involvement of additional photophysical processes unique to these systems under short-wavelength excitation.

It is important to note from the outset that the emission band observed in the 400–650 nm region is not a simple Stokes-shifted counterpart of the Soret band, as might initially be assumed. The Soret band corresponds to absorption from the ground state to the second excited singlet state (*S*_0_ → *S*_2_), *i.e.*, to high-energy electronic excitations involving π→π* transitions between molecular orbitals delocalized over the conjugated macrocyclic ring (HOMO-1 → LUMO).^40^ Direct fluorescence from S_2_ → S_0_ is usually quenched by fast internal conversion (on the fs–ps timescale),^41,42^ that's why the true Soret emission (*S*_2_ → *S*_0_) is essentially non-existent in most phthalocyanines. At the same time, as is known, Kasha's rule strongly favors emission from *S*_1_, therefore we tend to believe that the specified emission band has another origin and, most likely, results from a complex photophysical process associated with photoemis-sion of aggregates and deprotonation of the carboxyl groups upon short-wavelength excitation.

A similar dual emissive spectrum of TcPcZn, excited at 375 nm and with the peaks situated at 502 and 697 nm, was also detected in our previous research,^1,43^ as well as in the results reported by Kun Jia *et al.*, following research conducted on hyperbranched zinc phthalocyanines (HBZnPCs).^44^ Analyzing the fluorescence emission evolution of HBZnPCs as a function of the water content in DMF/H_2_O mixed solvents, these authors assumed that an effective fluorescent energy transfer channel can be switched on or off by controlling the aggregation behaviors that were promoted by water addition. The energy transfer from the blue-emission to the red-emission band was allowed when the HBZnPCs was monomeric in DMF, while this energy transfer channel was blocked when the H-aggregates were formed after increasing the amount of water in the solvent mixture up to 60%, thus ensuring the transition from dual fluorescent emission of HBZnPCs in DMF solvent to a single blue-emitting band in DMF/H_2_O (40/60) mixed solvent.

By carefully analyzing the preparation method of ZnP1–ZnP3 solutions described at the beginning in correlation with their absorption (Fig. 1a) and emission spectra (Fig. 8a–d), we conclude that in all our TcPcZn/TFA/H_2_O systems, both the monomeric form of TcPcZn and its J- and H-aggregates are present, the concentration of the latter increasing from ZnP1 to ZnP3. This conclusion is also corroborated by several works in the scientific literature.^2,45,46^ Also, resulting from the particularly high TFA : TcPcZn molar ratio in all these systems and the strength of the TFA (p*K*_a_ ∼0.2–0.5), we understand that in each of them, before excitation, TcPcZn is in its neutral form (due to protonation of the carboxyl groups) and not in the ionized one, and this fact, as mentioned above, favors aggregation. H-type aggregation blue-shifts absorption and quenches the normal Q-band fluorescence. Therefore, the emission observed in that 400–650 nm range upon short wavelength excitation (Fig. 8a–d) most likely originates from more photoactive, distorted or disordered J-aggregates,^44,47^ emitting from excited excimers or higher energy levels, while the weak emission band (750–850+ nm) of the same spectrum is the residual Q-band emission from unaggregated (monomeric) 
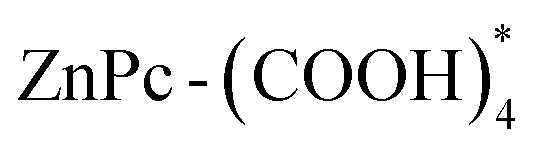
 or, more likely, from the unaggregated TcPcZn anions formed upon excitation.

**Fig. 9:**
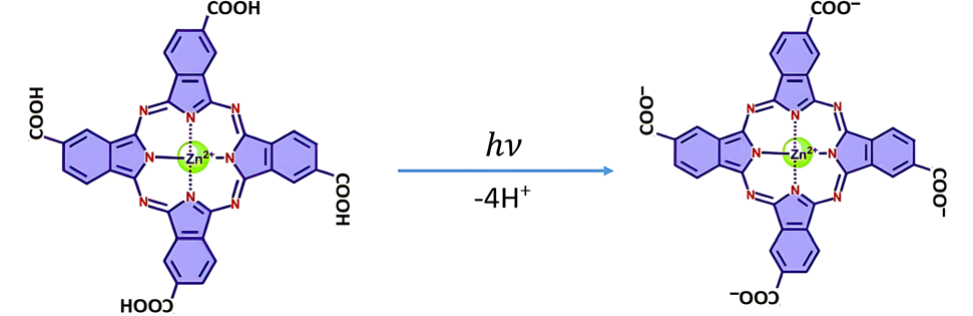
Scheme of the deprotonation of TcPcZn upon short wavelength excitation in water.

According to calculations, the standard free energy of dissociation of the carboxyl group of TcPcZn in water is between 0.23–0.29 eV. Therefore, the energy of short-wavelength radiation (390–360 nm ↔ 3.18–3.44 eV) is sufficient to cleave the O–H bond from the –COOH group and to trigger the deprotonation of all four carboxyl groups in the molecule upon excitation in water (Fig. 9). In our ZnP1–ZnP3 solutions, due to the low pH caused by the presence of TFA (see Table 1), the full deprotonation of the excited TcPcZn* molecules, even upon excitation at shorter-wavelengths, is highly unlikely, and only partial excited-state deprotonation may occur. At least 1–2 carboxyl groups are very likely to undergo excited-state proton transfer (ESPT) according to the schemes:
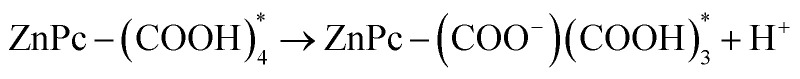

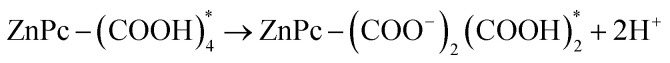


For the 3rd and 4th COOH groups, involvement in ESPT is possible but less probable due to strong hydrogen bonding, coulombic repulsion (if two or more are already deprotonated), and proton availability (low pH buffers ESPT reversibility). Deprotonation of TcPcZn destabilizes both H- and J-aggregates by introducing electrostatic repulsion and increasing solubility. Taking these factors into account, we assume that in the case of the ZnP3 solution, upon excitation at 355 nm (see Table 2), the longer fluorescence component with a lifetime of 3.79 ns arises from monomeric decay of the singlet excited state of TcPcZn, while the major component, with a lifetime of 0.67 ns, is attributed to emission from TcPcZn aggregates. Hence, a new biexponential fit is required to accurately analyze the fluorescence decay curve (Fig. 7b).

As mentioned above, and we can see again in the spectra shown in Fig. 8, excitation of the TcPcZn/TFA/H_2_O systems at longer wavelengths (660 and 705 nm) yields only the Stokes-shifted counterpart of the Q-band in the fluorescence spectra. This is because only monomeric or weakly aggregated TcPcZn absorbs efficiently at 660–705 nm, whereas H- and J-aggregates do not. This is probably due to exciton splitting in H-aggregates and the low or diffuse oscillator strength of J-aggregates.^38,48^ Theoretically, excitation of the systems even with this type of radiation can ensure the deprotonation of TcPcZn (705–660 nm ↔ 1.76–1.88 eV) and water molecules can act efficiently as temporary/transient proton acceptors in the mixed solution. Hence, the excited-state reaction model (ESR), involving partial dissociation of TcPcZn upon excitation, could also be applied in these cases, and one of the causes of the broadening of the Q-band fluorescence upon longer-wavelength excitation could be the coexistence of multiple excited species such as 
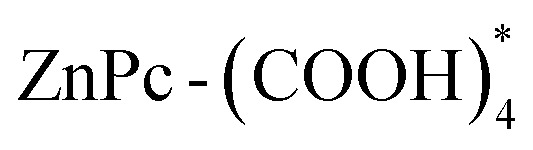
, 
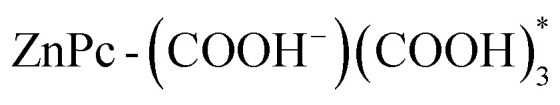
, and others in the excited systems.

Like in EP-PDI systems, the decrease in fluorescence quantum yield is attributed to pronounced solvent quenching, driven by specific solvent–fluorophore interactions. Hydrogen bonding promotes aggregation, which leads to self-quenching through exciton coupling or nonradiative energy transfer, decreasing quantum yield. Following excitation, the dipoles of H_2_O and TFA interact with that of TcPcZn and can reorient or relax around it, which strongly lowers the energy of the excited state of the fluorophore. Most likely, the lifetimes of the TcPcZn species could not be recorded in the more diluted ZnP1 and ZnP2 systems due to an increased rate of non-radiative decay processes (*k*_nr_) induced by the mixed solvent environment.

**Fig. 10:**
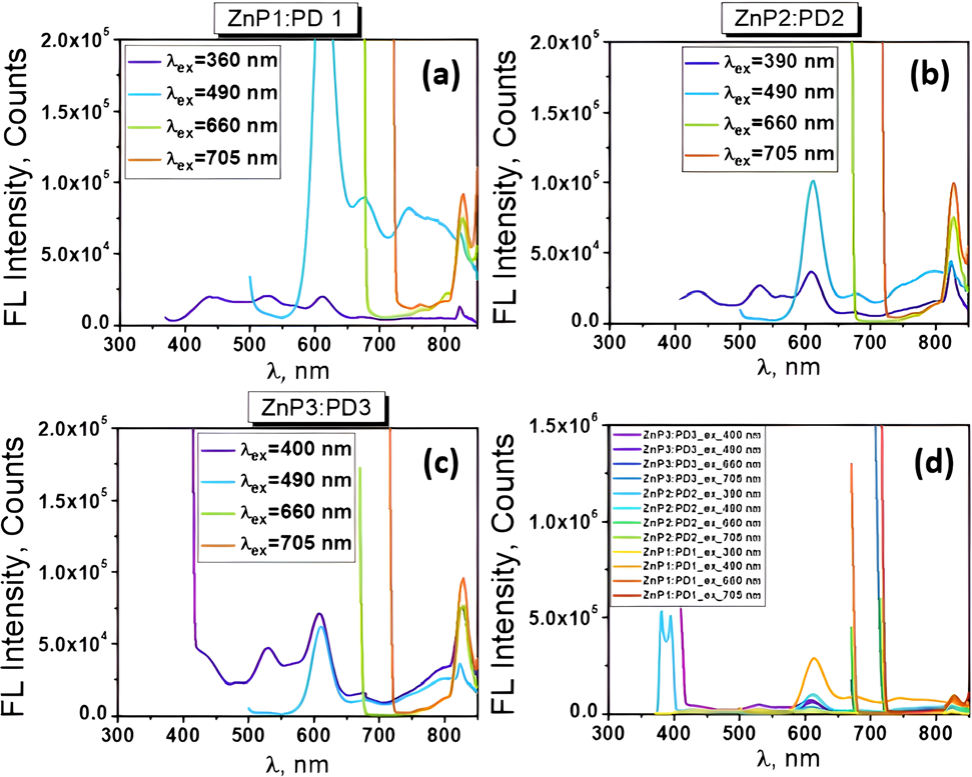
Fluorescence spectra of the TcPcZn : EP-PDI mixtures in the ZnP1 : PD1-ZnP3 : PD3 mixed solutions.

As expected, the most complex, but also the most interest-ing in terms of content, are the fluorescence spectra of the TcPcZn : EP-PDI mixed systems in TFA/H_2_O mixed solvents (Fig. 10a–d). As can be seen, the emission spectra of all these systems in ZnP1 : PD1-ZnP3 : PD3 mixed solutions, upon excitation at *λ*_ex_ = 660 nm and 705 nm, are practically identical to those of TcPcZn in ZnP1–ZnP3 solutions upon excitation at the same wavelengths (Fig. 8a–d). Each of them consistently exhibits a band starting at approximately 750 nm and extending beyond 850 nm, with a maximum around 828 nm. This spectral region can therefore be attributed to the excited state of TcPcZn (TcPcZn*) and likely to its previously mentioned anionic species, which coexist in these solutions upon excitation.

Particularly interesting are the fluorescence spectra of the same mixed systems upon excitation at 490 nm (see the celestial blue-colored graphs in Fig. 10). We observe that each of them exhibits an intense band between 550 and 650 nm, as well as a broader, unstructured band starting at approximately 650 nm and extending beyond 850 nm. By comparing these fluorescence spectra with those of EP-PDI (Fig. 6a–c) and TcPcZn (Fig. 8a–c) we conclude that the most intense band corresponds to the slightly blue-shifted emission of monomeric EP-PDI, while the broader, unstructured emission band can be considered a hallmark of a charge transfer (CT) state,^29,49,50^ an excimer, or an aggregated species. As can be seen, the intensity of both bands is higher in the case of the ZnP1 : PD1 solution and decreases proportionally with increasing concentrations of solute species in the other solutions subjected to excitation (Fig. 10a–c). The explanation lies in the fact that, in the more diluted ZnP1 : PD1 solution, the monomeric forms of TcPcZn and EP-PDI prevail, while the concentration of aggregates is lower. Given the electron-rich character of ZnPc(COOH)_4_,^2,51^ and the strong electron-accepting nature of EP-PDI,^6,7^ our emission observations strongly support the idea of photoinduced charge transfer between ZnPc-(COOH)_4_ (donor) and EP-PDI (acceptor). The formation of a charge transfer complex upon excitation is plausible, particularly in our polar and protic TFA/H_2_O media which promotes hetero-aggregation (TcPcZn–EP-PDI stacking, see Fig. 5). When the excited 
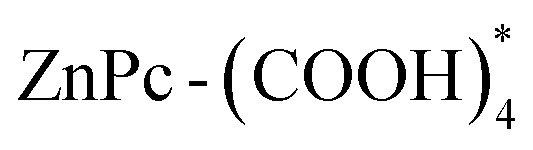
 and EP-PDI are in close proximity (*e.g.*, *via* π–π stacking, hydrogen bonding, or co-aggregation), photoinduced electron transfer can occur, according to the scheme: 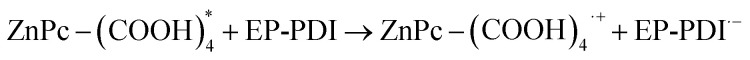


It should be noted that, while fully efficient photoinduced charge separation typically results in fluorescence quenching, weakly coupled donor–acceptor assemblies can form exciplex or partially charge-separated states that decay radiatively. Such relaxed CT/exciplex emission is characteristically broad, red-shifted, and structureless, consistent with the 650–850+ nm emission observed for the TcPcZn : EP-PDI mixtures. The polar/protic TFA/H_2_O medium likely stabilizes these relaxed CT/exciplex states, which aligns with the observed spectral shape and its dependence on concentration.

In the case of ZnP2 : PD2 and ZnP3 : PD3 solutions, the concentration of monomeric species is lower due to the presence of homoaggregates of TcPcZn and EP-PDI, which were formed in the individual ZnP2, ZnP3, PD2, and PD3 solutions prior to mixing for the preparation of the mixed systems. This contributes to a decreased probability of heteroaggregate formation (and consequently, of photoinduced charge transfer upon excitation at *λ*_ex_ = 490 nm), as well as to a reduction in the intensity of the aforementioned emission bands.

**Fig. 11:**
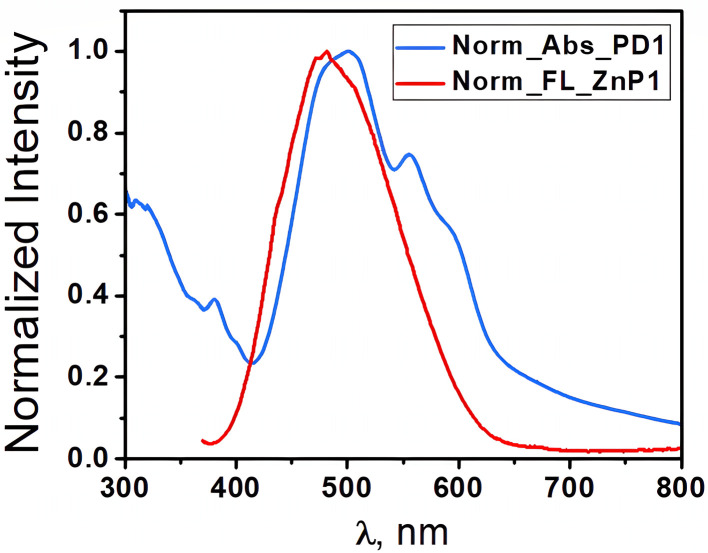
Normalized emission spectra of TcPcZn (*λ*_ex_ = 360 nm) and normalized absorption spectra of EP-PDI in TFA/H_2_O mixed solvent.

Also, highly informative and noteworthy are the emission spectra of the same mixed systems upon excitation at *λ*_ex_ ≤ 400 nm. As shown in Fig. 10a, the fluorescence spectrum of the TcPcZn : EP-PDI system in the ZnP1 : PD1 solution upon excitation at 360 nm is strongly quenched and consists of a broad, unstructured band of very low intensity in the 380–650 nm range, with maxima at 435, 530, and 610 nm, as well as an even weaker band in the 700–850+ nm region, peaking at 823 nm. The exceptionally low intensity of this spectrum can be attributed to several factors. First, as mentioned earlier, the monomeric species of TcPcZn and EP-PDI prevail in the mixed ZnP1 : PD1 solution due to the higher degree of dilution. As is already known, the photoemission of these monomeric species upon excitation at these wavelengths is strongly quenched, especially due to specific solvent–fluorophore interactions. Secondly, in this solution, the concentration of aggregates of both solute types is significantly reduced after mixing equal volumes of ZnP1 and PD1 solutions to prepare the ZnP1 : PD1 mixture. Moreover, as can be seen, the emission band in the 400–650 nm region, previously attributed to the photoemission of photoactive TcPcZn aggregates (see Fig. 8a and 10), is strongly attenuated in this spectrum. This is due, on the one hand, to the reduced concentration of aggregates, and, on the other hand, to a possible Förster Resonance Energy Transfer (FRET) mechanism. This non-radiative energy transfer is very likely to occur between the photoactive aggregates of TcPcZn and the monomeric species of EP-PDI upon excitation. As shown in Fig. 11, the emission spectrum of TcPcZn upon excitation at shorter wavelengths overlaps with the absorption spectrum of EP-PDI in the 400–650 nm region, fulfilling a key requirement for FRET to occur.

The fluorescence spectrum of the TcPcZn : EP-PDI system in the ZnP2 : PD2 mixed solution, upon excitation at 390 nm, is shown in Fig. 10b. It exhibits higher intensity and more pronounced spectral structuring compared to the previous spectrum. Also, we observe that the peak positions remain unchanged both in the broad band within the 400–650 nm region and in the one from the 700–850+ nm range. The observed increase in intensity of the broad emission band is due to the elevated concentration of photoactive J-aggregates of TcPcZn in the mixed solution, while its structuring is likely the result of specific interactions between these aggregates and EP-PDI species. The broadening and increased intensity of the band in the 700–850+ nm range may be attributed to a combination of factors, such as a change in the excitation wavelength, photoinduced charge transfer (CT) between TcPcZn and EP-PDI species, and possibly others.

In Fig. 10c, the violet-colored graph represents the fluorescence spectrum of the TcPcZn : EP-PDI system in the ZnP3 : PD3 mixed solution, upon excitation at 400 nm. This spectrum is very similar to the previously analyzed one; however, its intensity is nearly twice as high, likely due to increased concentrations of all species present in the solution. We also observe that two peaks remain at the same positions (at 530 nm and around 610 nm), while the peak in the longer-wavelength region is red-shifted by 3 nm, which indicates a slight enhancement of photoinduced CT between the monomeric species in the solution upon excitation. We also note a distortion of the band with the peak at 435 nm, which we attribute to FRET from the photoactive TcPcZn aggregates—whose concentration in the current solution is higher than in the previous one—to the EP-PDI species upon excitation. This assumption is based on the observation of decreased lifetime values attributed to emission from TcPcZn aggregates, from 0.67 ns in the ZnP3 solution to 0.64 ns in the ZnP3 : PD3 mixed solution (see Table 2).

By applying the formula:1
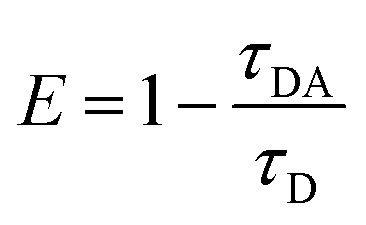
where *τ*_D_ is the ensemble-average donor fluorescence lifetime in the absence of the acceptor, and *τ*_DA_ is the ensemble average donor fluorescence lifetime in the presence of the acceptor, we estimate an effective average FRET efficiency (*E*) of approximately 5% in our system.

As is well known, eqn (1) is primarily applicable to donor–acceptor (D–A) pairs separated by a fixed distance, a situation commonly encountered in labeled proteins.^36,52,53^ However, a single, well-defined D–A distance does not apply to systems consisting of a mixture of donors and acceptors in solution, nor to randomly dispersed donors and acceptors within membranes. In such cases, more complex expressions are required, typically derived by averaging the energy transfer rate over the assumed spatial distribution of donor–acceptor pairs.^54–56^

Nevertheless, eqn (1) remains widely used in complex systems where detailed structural resolution is not available— such as in biological membranes, polymer blends, and nanoparticle–dye aggregates.^57–61^

Based on these considerations, we attribute the reduced fluorescence quantum yield observed in our mixed systems — compared to the individual components (see Table 2) — to the coexistence of two distinct processes. In the 400–650 nm region, a modest decrease in the short-lived TcPcZn aggregate lifetime (*τ*_1_ = 0.67 → 0.64 ns at *λ*_ex_ = 355 nm) and the partial attenuation of the donor emission suggest a weak FRET contribution from TcPcZn aggregates to monomeric EP-PDI. In contrast, the pronounced decrease in quantum yield at longer wavelengths (from 9.51% in the ZnP3 solution to 1.99% in the ZnP3 : PD3 mixture) arises from the formation of exciplex- or partially charge-separated CT states stabilized by the polar/protic TFA/H_2_O medium, whose weak radiative relaxation coexists with dominant nonradiative decay pathways, leading to an overall reduction in *Φ*.

To provide a broader perspective on the photophysical behavior, we analyzed the overall correlation between fluorescence lifetimes and quantum yields across all investigated systems. The observed trend follows the fundamental photophysical relationship, where longer excited-state lifetimes are generally associated with higher quantum yields, reflecting a lower contribution from non-radiative decay pathways. This correlation is also consistent with the specific quenching mechanisms discussed above — such as aggregation, excimer formation, and solvent-induced relaxation — which enhance non-radiative processes and consequently reduce both *τ* and *Φ*. For instance, EP-PDI in the PD3 system exhibits an average (amplitude-weighted) lifetime of *τ*_avg_ ≈ 1.78 ns with *Φ* ≈ 6.0%, whereas TcPcZn (ZnP3, *λ*_ex_ = 660 nm) shows a longer *τ*_avg_ ≈ 3.79 ns and a higher yield (*Φ* ≈ 9.5%). In contrast, the TcPcZn : EP-PDI mixed system (ZnP3 : PD3) displays reduced lifetimes (*τ*_1_ = 0.64 ns, *τ*_2_ = 3.96 ns; *τ*_avg_ ≈ 1.82 ns) and a lower quantum yield (*Φ* ≈ 1.9%), consistent with additional non-radiative decay channels, most likely involving FRET and charge-transfer interactions. Overall, these results demonstrate that longer lifetimes correspond to higher emission efficiencies, whereas quenching processes in the mixed systems simultaneously decrease both *τ* and *Φ*.

**Fig. 12:**
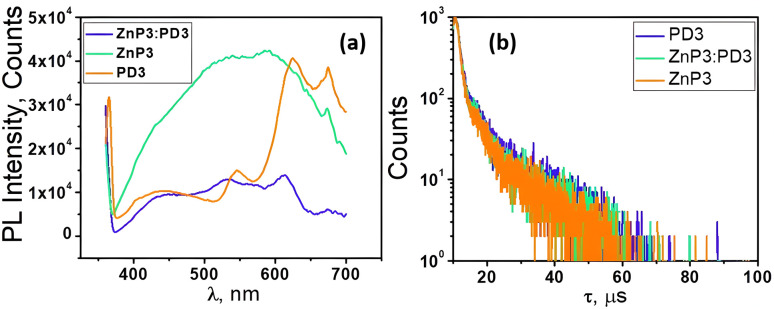
(a) Phosphorescence (PL) spectra of TcPcZn in ZnP3, EP-PDI in PD3, and TcPcZn : EP-PDI mixture in ZnP3 : PD3 mixed solutions; (b) phosphorescence lifetime spectra of TcPcZn, EP-PDI, and the TcPcZn : EP-PDI self-assembled system in a TFA/H2O mixed solvent.

According to Fig. 12a, both the individual compounds TcPcZn and EP-PDI, as well as their mixtures in TFA/H_2_O mixed solvents, exhibit room-temperature phosphorescence. As shown, the RTP intensities are of the same order of magnitude, though slightly higher in the individual systems.

Table 3 presents the triplet excited-state lifetime values of the TcPcZn and EP-PDI compounds, as well as those of their TcPcZn : EP-PDI mixture, measured in a TFA/H_2_O mixed solvent system upon excitation at 355 nm. The measured phosphores-cence lifetimes for TcPcZn (1.21 µs and 8.93 µs) are consistent with those reported in our previous work.^1,43^

**Table 3:** Phosphorescence lifetimes (*τ*_1_ and *τ*_2_) and relative contributions (% Rel)

Samples	Ph			
	*τ*_1_, µs	*τ*_2_, µs	Rel *τ*_1_, %	Rel *τ*_2_, %
PD3	1.32	10.18	60.98	39.02
ZnP3 : PD3	1.15	9.41	60.01	39.99
ZnP3	1.21	8.93	65.15	34.85

To our knowledge, no prior studies have reported the room temperature phosphorescence lifetime of EP-PDI. Therefore, the values presented here (1.32 µs and 10.18 µs) represent the first documented RTP lifetimes for this compound.

The triplet excited-state lifetime of the TcPcZn : EP-PDI mixture, like those of the individual compounds, exhibits a bi-exponential decay kinetics (Fig. 12b). The observed intermediate lifetimes (1.15 µs and 9.41 µs) suggest an interaction involving energy transfer (most likely FRET and/or PET) between ZnPc-(COOH)_4_ and EP-PDI that outcompetes intersystem crossing, thereby reducing the triplet state population.

We attribute the inability to measure phosphorescence quantum yields primarily to the solvent environment, which inherently favors decay through non-phosphorescent pathways. Although transient hydrogen bonding may locally stabilize molecular conformations, the low viscosity and dynamic nature of the TFA/H_2_O environment allow TcPcZn and EP-PDI molecules to retain substantial vibrational and rotational freedom, which, combined with frequent molecular collisions, favors non-radiative triplet decay over phosphorescence.

Additionally, being exposed to air, dissolved oxygen is abundant in our aqueous-acidic solutions and rapidly quenches triplet states through energy transfer.^62,63^ Moreover, the afore-mentioned interactions between TcPcZn and EP-PDI in the mixed systems create new competitive decay pathways that bypass the triplet states entirely or rapidly deplete them.

To achieve higher RTP quantum yields, a series of measures must be undertaken. The most important among these include: (i) rigidifying the environment by embedding the phosphors in solid matrices such as poly(methyl methacrylate) (PMMA), chitosan or silica;^1,2,64^ (ii) eliminating oxygen (O_2_) by purging the solutions with N_2_ or Ar, using airtight sample cells or glove-box preparation, and performing storage and measurements under an inert atmosphere;^65,66^ (iii) enhancing intersystem crossing (ISC) by incorporating heavy atoms (*e.g.*, Br or I) into the phosphor molecules.^36,67^

## Conclusions

Absorption and fluorescence spectroscopy suggest that both TcPcZn and EP-PDI form highly dynamic aggregates through self-assembly in TFA/H_2_O mixtures, primarily driven by high concentration. The tendency of these compounds to self-assemble in solutions arises from π–π stacking interactions enabled by their aromatic systems. In the case of TcPcZn, this process is also facilitated by the protonation of the carboxyl groups.

Fluorescence spectra of the mixed systems, excited at 490 nm, confirmed that co-assembling TcPcZn with EP-PDI in TFA/H_2_O mixtures promotes favourable donor–acceptor inter-actions, resulting in enhanced light absorption and emission properties. These interactions were found to be most efficient in solutions where monomeric species of TcPcZn and EP-PDI prevail, while an optimal distance between them is maintained. Therefore, for practical applications, careful adjustment of so-lute concentrations is essential.

Specific interactions between TcPcZn, EP-PDI, and their co-aggregated forms with the TFA/H_2_O mixture and the abundance of molecular oxygen in the studied solutions are the main factors contributing to the quenching of the triplet states.

## Author contributions

Dr Tamara Potlog: conceptualization, methodology, funding acquisition, supervision. Dr Vadim Furtuna: investigation, data curation, formal analysis, interpretation of spectral data, writing – original draft. Dr Ion Lungu: software development, construction of all figures, consultation. PhD student Alexandrina Druta: consultation, English *trans*-lation, visualization. Prof. Anton Airinei: validation, supervision. All authors have read and approved the final manuscript.

## Conflicts of interest

There are no conflicts to declare.

## Data Availability

The experimental data supporting the findings of this study are available from the corresponding author upon reasonable request.
